# Exploration of the total-body PET/CT reconstruction protocol with ultra-low ^18^F-FDG activity over a wide range of patient body mass indices

**DOI:** 10.1186/s40658-022-00445-3

**Published:** 2022-03-03

**Authors:** Xiuli Sui, Hui Tan, Haojun Yu, Jie Xiao, Chi Qi, Yanyan Cao, Shuguang Chen, Yiqiu Zhang, Pengcheng Hu, Hongcheng Shi

**Affiliations:** 1 Department of Nuclear Medicine, Zhongshan Hospital, Fudan University, 180 Fenglin Road, Shanghai, 200032 China; 2 Nuclear Medicine Institute of Fudan University, Shanghai, 200032 China; 3 Shanghai Institute of Medical Imaging, Shanghai, 200032 China

**Keywords:** Total-body PET/CT, Ultra-low activity, Image quality, Reconstruction, BMI

## Abstract

**Purpose:**

The purpose of this study was to investigate the image quality and diagnostic performance of different reconstructions over a wide range of patient body mass indices (BMIs) obtained by total-body PET/CT with ultra-low ^18^F-FDG activity (0.37 MBq/kg).

**Methods:**

A total of 63 patients who underwent total-body PET/CT with ultra-low activity (0.37 MBq/kg) ^18^F-FDG were enrolled. Patients were grouped by their BMIs. Images were reconstructed with the following two algorithms: the ordered subset expectation maximization (OSEM) algorithm (2, 3 iterations), both with time of flight (TOF) and point spread function (PSF) corrections (hereinafter referred as OSEM2, OSEM3) and HYPER Iterative algorithm (*β*-values of 0.3, 0.4, 0.5, 0.6) embedded TOF and PSF technologies (hereinafter referred as HYPER0.3, HYPER0.4, HYPER0.5 and HYPER0.6, respectively). Subjective image quality was assessed by two experienced nuclear medicine physicians according to the Likert quintile, including overall image quality, image noise and lesion conspicuity. The standard deviation (SD) and signal-to-noise ratio (SNR) of the liver, and maximum standard uptake value (SUV_max_), peak standard uptake value (SUV_peak_), tumour background ratio (T/N) and the largest diameter of lesions were quantitatively analysed by a third reader who did not participate in the subjective image assessment.

**Results:**

Increased noise was associated with increased BMI in all reconstruction groups. Significant differences occurred in the liver SNR among BMI categories of OSEM reconstructions (*P* < 0.001) but no difference was seen in the HYPER Iterative reconstructions between any of the BMI categories (*P* > 0.05). With the increase in BMI, overall image quality and image noise scores decreased significantly in all reconstructions, but there was no statistically significant difference of lesion conspicuity. The overall image quality score of the obese group was not qualified (score = 2.7) in OSEM3, while the others were qualified. The lesion conspicuity scores were significantly higher in HYPER Iterative reconstructions and lower in OSEM2 than in OSEM3 (all *P* < 0.05). The values of SUV_max_, SUV_peak_ and T/N in HYPER0.3, HYPER0.4 and HYPER0.5 were higher than those in OSEM3. In different reconstructions, there was a correlation between lesion size (median, 1.55 cm; range, 0.7–11.0 cm) and SUV_peak_ variation rate compared to OSEM3 (r = 0.388, − 0.515, − 0.495, − 0.464, and − 0.423, respectively, and all *P* < 0.001).

**Conclusion:**

Considering the image quality and lesion analysis in ^18^F-FDG total-body PET/CT with ultra-low activity injection, OSEM reconstructions with 3 iterations meet the clinical requirements in patients with BMI < 30. In patients with BMI ≥ 30, it is recommended that the HYPER Iterative algorithm (*β*-value of 0.3–0.5) be used to ensure consistent visual image quality and quantitative assessment.

## Introduction

Current clinical standard axial FOV PET/CT scanners cover an axial range of 15–30 cm which requires 5–9 bed positions to acquire the whole-body PET images. Standardized activity (3.5–3.8 MBq/kg) of ^18^F-FDG was administered intravenously, and approximately 2–4 min/bed was need to achieve diagnostic image quality, taking almost a 10–20 min acquisition time for whole-body PET images [1–3]. Recently, several long axial FOV (LAFOV) devices have arisen, such as uEXPLORER (AFOV of 194 cm), Quadra (AFOV of 106 cm) and PennPET (AFOV of 64 cm) [4–6]. A total-body PET scanner (uEXPLORER) with an axial field of view of 194 cm was used to provide up to 40 times the effective count rate than ~ 22 cm AFOV scanners for total-body applications [4, 7]. For single-organ imaging, gain can be used to acquire diagnostic PET images with very small amounts of activity in the field of view [8]. Theoretically, the effective count rate of using 10× reduction injected activity in LAFOV scanners is much higher than that of conventional scanners [4, 9].

In dose-reduction research, previous results have shown that high quality images being achieved after 25 MBq activity injection (0.57 MBq/kg) scanned over 10 min (reconstructed using OSEM-PSF-TOF) in one subject (43.5 kg, 152 cm) [8]. Our team has also shown that total-body PET with half-dose ^18^F-FDG activity (1.85 MBq/kg) over 2–4 min scans could achieve a comparable image quality to conventional PET, and the image quality was even superior to that of conventional PET [10]. Recent research also demonstrated that total-body dynamic PET imaging using a 10 × reduction in injected activity achieved comparable image contrast with full-activity imaging [11]. The image quality of ultra-low activity (0.37 MBq/kg) with 7–15 min scan duration is sufficient for diagnosis [12]. Optimal image quality could be achieved with a simulated administered dose-reduction down to 0.37 MBq/kg in pediatric patients [13]. More cases are required to verify the sensitivity and accuracy of this approach in adults.

Ordered subset expectation maximization (OSEM) is the most commonly used image reconstruction method in PET clinical practice. The mean feature of OSEM is that the noise increases with the number of iterations in which image is unacceptable for clinical purpose when the count rate is relatively low. If 1 or 2 iterations are performed, the contrast recovery is insufficient, and the lesions cannot be well displayed. Therefore, a trade-off needs to be made between image noise and quantitative accuracy, which results in insufficient image convergence [14, 15]. High-quality images can be reconstructed using the OSEM algorithm over a 15-min scan with a 10× reduction in injected activity in the LAFOV scanner. However, the noise-equivalent count rate (NECR) fell rapidly with increasing weight [16], and the effects of body mass index (BMI) on NECR and image noise also showed that patients with a larger BMI consistently reconstructed poor image quality when using OSEM algorithm [17]. The image quality reconstructed using OSEM may not be able to determine the clinical diagnosis, and there is a necessary to find an effective reconstruction algorithm to improve the image quality to obtain qualified images. A new Bayesian penalized likelihood reconstruction algorithm (HYPER Iterative) developed by United Imaging compensates for this shortcoming [18]. The HYPER Iterative algorithm incorporates noise control into each iteration, and finds the maximum likelihood solution through repeated iterations. Thus, the image can significantly suppress noise while achieving optimal convergence.

Based on these above explorations, the purpose of our study is to analyse the image quality of different reconstructions obtained by total-body PET/CT with ultra-low ^18^F-FDG activity (0.37 MBq/kg) over a wide range of patient body mass indices.

## Materials and methods

### Patients’ selection and image acquisition

This study was approved by the Medical Ethics Committee of Zhongshan Hospital Affiliated to Fudan University (2019-029R), and informed consent was obtained. Patients who underwent total-body ^18^F-FDG PET/CT with ultra-low activity injection (0.37 MBq/kg) at Zhongshan Hospital Fudan University from January, 2020 to June, 2021 were analysed retrospectively. Patients with malignant tumours who providing histological confirmation, were eligible for analysis. Patients were excluded if they had no FDG avid findings. Eventually, 63 patients with weights ranging from 38 to 110 kg who had a baseline preoperative ^18^F-FDG PET/CT scan with list mode data available for reconstruction were included in this study. Patients were then grouped by their BMI (weight in kilograms divided by the square of height in metres) categories according to the criteria of the WHO [19]: underweight (BMI ≤ 18.5), normal (BMI 18.5–24.9), overweight (BMI 25–29.9) and obese (BMI ≥ 30).

Patients were required to fast for at least 6 h and avoid strenuous exercise prior to ^18^F-FDG PET/CT imaging. The fasting blood glucose level was less than 7.0 mmol/L. Patients received an injection of ^18^F-FDG according to their body weight (0.37 MBq/kg). ^18^F-FDG with more than 95% radiochemical purity was provided by Shanghai Atom Kexing Pharmaceutical Co. Ltd. All patients rested quietly for approximately 60 min after the injection of ^18^F-FDG and then underwent PET/CT imaging. List mode PET data were acquired for 15 min using a total-body PET/CT scanner (uEXPLORER, United Imaging Healthcare, Shanghai, China).

### Image reconstruction

Raw data of each patient were reconstructed using two algorithms: OSEM and HYPER Iterative (United Imaging Healthcare, Shanghai, China) [20, 21]. In HYPER Iterative, the penalized likelihood function is written as follows:

1 $$\hat{f} = \arg \max_{f \ge 0} \left[ {\sum\limits_{ij} { - p_{ij} f_{j} } + \sum_{i} c_{i} {\text{ln}}\left( {\sum_{j} p_{ij} f_{j} } \right) - \sum_{j} \gamma_{j} \cdot U\left( {f_{j} } \right)} \right]$$

2 $$\gamma_{j} = g\left( {NEC, sns_{j} } \right) \cdot \beta$$

3 $$U\left( f \right) = \sum_{x,y,z} \left| {\nabla f} \right|$$

where *i* and *j* are the indexes of the projection bins and image pixels, respectively. *f* is the image estimate. $c_{i}$, are the measured emission data. $p_{ij}$ is the system matrix indicating the counts emitted from the *j*th image pixel detected by the *i*th projection bin. *β* is a factor representing penalty strength which is normalized to a range of 0.01 to 1.00. $\gamma_{j}$ is a parameter of regularized strength. $U$ is the total variation penalization of the pixels in the neighborhood. NEC denotes the noise equivalent counts. $sns_{j}$ is the spatially varied sensitivity profile. $g$ is a function of NEC and $sns_{j}$.

In HYPER Iterative, time of flight (TOF) and point spread function (PSF) modelling were included and no postfilter was applied. These options, as well as the iteration number, were pre-defined by the manufacturer, and they are not allowed to be changed by the users. Penalty strength *β* is the only adjustable parameter provided to the user that can be adjusted in the interval of (0, 1]. The penalty strength *β* controls the smoothness of the reconstructed image. Larger *β* provides smoother images.

In the pre-analysis of 5 patients, raw datasets were reconstructed using 8 different reconstruction options: OSEM reconstructions (2, 3 and 4 iterations), and HYPER Iterative reconstructions (extensive *β*-values of 0.1, 0.3, 0.5, 0.7 and 1, respectively). We observed that HYPER Iterative reconstructions (*β*-values of 0.3, 0.5) and OSEM reconstruction (2, 3 iterations) have good image quality and lesion conspicuity. The groups with β value of 0.7 and 1 were rejected due to their blurring effects (data not shown). For further detailed analysis, *β*-values had to be adjusted to improve the image quality. The datasets were reconstructed using the HYPER Iterative algorithm (embedded TOF and PSF technologies with no post filter) with *β*-values of 0.3, 0.4, 0.5, and 0.6 (hereinafter referred as HYPER0.3, HYPER0.4, HYPER0.5, HYPER0.6, respectively) and the OSEM algorithm with TOF and PSF which were later substituted as OSEM2 and OSEM3 (2 and 3 iterations, 20 subsets, and the full width at half maximum of the Gaussian filter function 3 mm). All reconstructions have the same matrix as follows: 192 × 192, FOV = 600, and slice thickness 1.443 mm (voxel grid 3.125 × 3.125 × 1.443 mm^3^). Standard corrections, including decay, random, dead time, attenuation and normalization correction, were applied in all PET reconstructions. The CT scan parameters were as follows: tube voltage 120 kV, tube current 140 mAs, pitch 1.0, collimation 0.5 mm, and reconstructed slice thickness 0.5 mm.

### Image analysis

The PET/CT images were independently evaluated by two experienced nuclear radiologists. According to the Likert quintile, the image quality was scored in 3 perspectives including overall image quality, image noise and lesion conspicuity: score 5, excellent diagnostic image quality, optimal noise, sharp lesion depiction, and free of the artefact, providing diagnosis with full confidence; score 4, image with quality that is superior to the average image quality; score 3, image with quality that is equivalent to those used in clinical practice; score 2, image with sub-optimal noise, lesion depiction leading to impaired diagnostic confidence; score 1, image with nondiagnostic quality, excessive noise, or unfavorable lesion contrast. A score of 3, 4, or 5 was considered to provide diagnostic value [22], indicating that the needs for clinical diagnosis could be met, whereas image quality scores of 1–2 did not meet the needs of clinical diagnosis.

**Fig. 1:**
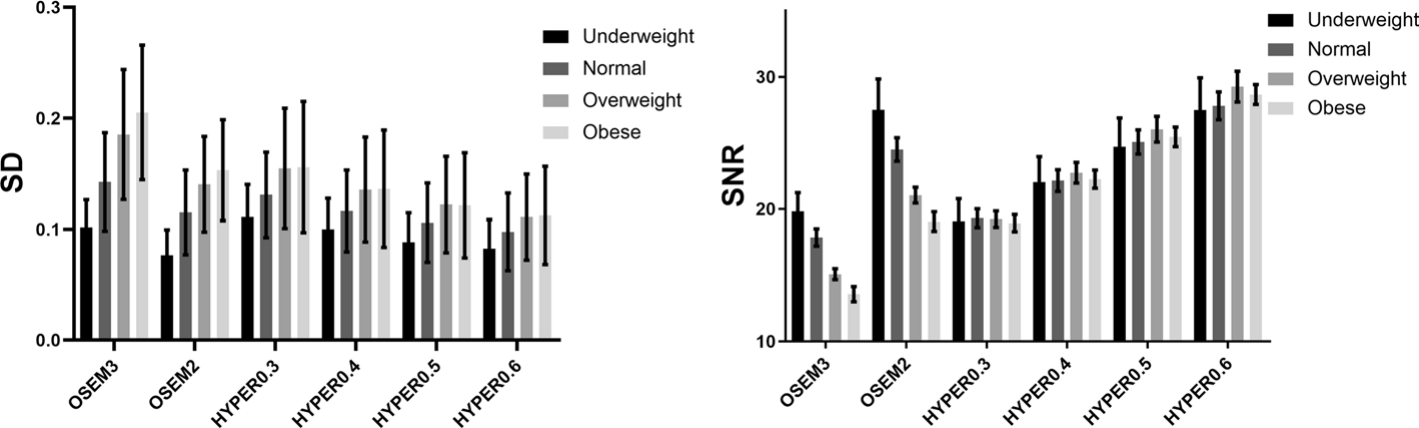
The bar graph of the SNR and noise in liver of different reconstructions

The mean standard uptake value (SUV_mean_) and SD of the liver, and maximum standard uptake value (SUVmax), peak standard uptake value (SUVpeak), tumour background ratio (T/N) and the largest diameter of lesions were measured by a third reader who did not participate in the subjective image assessment. Round-shaped (1 cm diameter) regions of interest (ROIs) was placed in four homogeneous area of the liver (avoiding intrahepatic lesions and larger blood vessels) to measure SUV_mean_ and SD, and one ROI was placed in the descending aorta to measure SUV_mean_. The spherical volumes of interest (VOIs) were placed on each lesion to measure SUV_max_ and SUV_peak_. The ROIs of all reconstructed images was drawn synchronously in 2D mode to ensure that each ROI was the same location and size. SD was defined as the noise, and the SNR is calculated by dividing the SUV_mean_ in the liver by its SD. All pathologically confirmed positive lesions on PET were analysed, with a maximum of 6 lesions selected per patient. If there were more than 6 lesions in one patient, 6 target lesions (three maximum and three minimum FDG-avid lesions) were defined for further analysis. Lesion size was defined as the largest diameter of lesions. The T/N was calculated by dividing the lesion SUV_max_ by the SUV_mean_ of the descending aorta. The variation rate of SUV_max_, SUV_peak_ and T/N (represented as ΔSUV_max_, ΔSUV_peak_ and ΔT/N in the rest of this paper) were calculated as values of (SUV_max_, SUV_peak_ and T/N in each reconstruction minus SUV_max_, SUV_peak_ and T/N in OSEM3) divide by SUVmax, SUVpeak and T/N in OSEM3, respectively [23] (Fig. 1).

### Statistical analysis

Microsoft Excel and SPSS 22.0 Windows software (IBM SPSS Inc., Armonk, New York, USA) were used for statistical analysis. Continuous variables are expressed as the mean ± standard deviation. Since the objective image quality of each reconstruction was distributed normally, significant differences were assessed using repeated measures analysis of variances (ANOVA) with post hoc Bonferroni corrections to adjust for multiple comparisons. Qualitative image ratings and quantitative SUV_max_, SUV_peak_ and T/N values of BMI groups were analyzed with Kruskal–Wallis test separately. Weighted kappa was used to test the consistency among the subjective rating individuals. The differences in SUV_max_, SUV_peak_, and T/N between reconstructions of each patient were calculated, and the mean value and standard deviation of these differences were then calculated for all patients. Linear regression was performed on the relationship between lesion size and ΔSUV_max_, ΔSUV_peak_ and ΔT/N. A *P* < 0.05 was taken to be significant.

## Results

### Patient characteristics

In this study, 63 patients with 94 lesions were enrolled as follows: 21 females (33.3%) and 42 males (66.7%) with an average age of 61.4 years (age range 21–81 years). There were a total of 18 cancer types, that were unevenly distributed. The lesion locations of the enrolled patients included the head and neck (n = 5), chest (n = 12), abdomen (n = 51), and pelvis (n = 26). There was no significant difference in the performance of the reconstruction methods for different locations (*P* > 0.05). There were no significant differences in age, sex, blood glucose level, uptake time or clinical stages among the four groups (all *P* > 0.05). Details for each BMI category are listed in Table 1.

**Table 1:** Clinical data and demographic of patients (n = 63) who underwent ^18^F-FDG total-body PET/CT with ultra-low activity injection

Characteristic	Underweight (n = 12)	Normal (n = 20)	Overweight (n = 20)	Obese (n = 11)	P
Age (years)	56.7 ± 17.3	62.1 ± 12.8	63.3 ± 12.6	61.8 ± 7.9	0.77
Sex					0.30
Male	8	12	12	10	
Female	4	8	8	1	
BMI (kg/m^2^)	17.2 ± 0.6	22.8 ± 1.8	26.6 ± 1.4	31.4 ± 1.4	< 0.0001
Blood glucose before injection (mmol/L)	5.6 ± 0.7	5.7 ± 0.9	6.1 ± 1.3	6.0 ± 0.7	0.53
Injected dose (MBq)	18.6 ± 1.9	24.0 ± 3.0	27.4 ± 4.0	34.2 ± 5.1	< 0.0001
Acquisition time (min)	62.8 ± 9.6	63.1 ± 7.5	63.3 ± 9.4	60.8 ± 9.6	0.87
Clinical stages					0.54
I	2	6	3	2	
II	4	5	3	2	
III	2	5	8	4	
IV	4	4	6	3	

### Image analysis

Increased noise was associated with increased BMI in all reconstruction groups. In OSEM reconstructions, significant differences in liver SNR were found among BMI categories (*P* < 0.001). In contrast, there was no significant difference in the liver SNR of HYPER Iterative reconstructions between any of the BMI categories (*P* > 0.05). The detailed results are listed in Table 2.

**Table 2:** Image quality using different reconstruction parameters

Reconstructions	Underweight (n = 12)		Normal (n = 20)		Overweight (n = 20)		Obese (n = 11)	
	SNR	Noise	SNR	Noise	SNR	Noise	SNR	Noise
OSEM3	19.8 ± 4.9	0.11 ± 0.03	16.8 ± 2.7	0.17 ± 0.04	15.0 ± 2.2	0.18 ± 0.02	12.7 ± 1.7	0.23 ± 0.04
OSEM2	27.5 ± 8.1**	0.08 ± 0.02**	23.1 ± 3.9**	0.12 ± 0.03**	21.0 ± 2.9**	0.13 ± 0.02**	18.1 ± 2.1	0.16 ± 0.03
HYPER0.3	19.1 ± 5.9	0.12 ± 0.03	19.7 ± 2.7	0.14 ± 0.03**	19.5 ± 3.2	0.14 ± 0.02**	18.6 ± 2.1	0.16 ± 0.03
HYPER0.4	22.0 ± 6.7	0.10 ± 0.02	23.0 ± 3.2**	0.12 ± 0.02**	23.0 ± 3.8**	0.12 ± 0.02**	21.9 ± 2.6**	0.13 ± 0.02**
HYPER0.5	24.7 ± 7.6*	0.09 ± 0.02	26.4 ± 4.0**	0.11 ± 0.02**	26.1 ± 4.5**	0.11 ± 0.02**	25.4 ± 3.0**	0.11 ± 0.02**
HYPER0.6	27.5 ± 8.5**	0.08 ± 0.02**	29.4 ± 4.6**	0.10 ± 0.02**	29.3 ± 5.2**	0.09 ± 0.02**	28.7 ± 3.2**	0.10 ± 0.02**

Data are means ± standard deviations, *P < 0.05, **P < 0.01

**Fig. 2:**
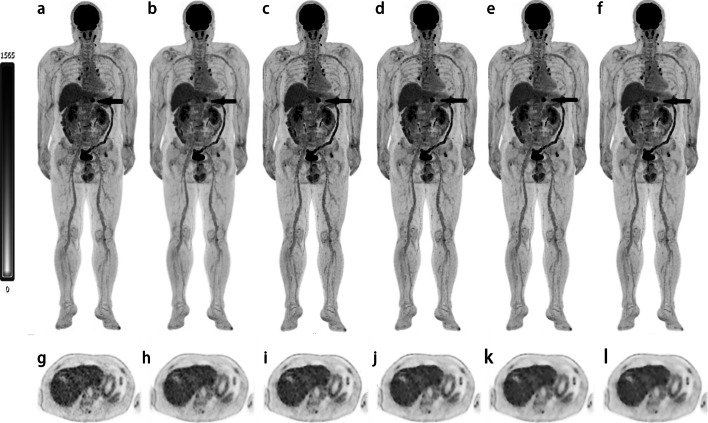
Selections of MIPs and transaxial view images from reconstructions of a 62-year-old man with pancreatic cancer confirmed by surgery (**a**–**l**) for OSEM3 (**a**), OSEM2 (**b**), HYPER0.3 (**c**), HYPER0.4 (**d**), HYPER0.5 (**e**), HYPER0.6 (**f**) and OSEM3 (**g**), OSEM2 (**h**), HYPER0.3 (**i**), HYPER0.4 (**j**), HYPER0.5 (**k**), HYPER0.6 (**l**). The black arrows show avid FDG of the pancreas. The overall image scores of 2, 3, 3, 3, 4, and 4 were given to the 6 groups respectively

The interrater agreement for the image quality score was excellent (weighted kappa = 0.827, 95% confidence interval, 0.801–0.854). With the increase in BMI, overall image quality and image noise scores decreased in the OSEM3, OSEM2, HYPER0.3, HYPER0.4, HYPER0.5 and HYPER0.6 groups. When lesion conspicuity scores were compared, there were no significant differences among the four groups. The overall image quality scores of the underweight, normal and overweight groups were qualified (3+) in all reconstructions, while the obese group in OSEM3 was not (2.7). Similar differences were observed in image noise. The lesion conspicuity scores were significantly higher in HYPER Iterative reconstructions and lower in OSEM2 than in OSEM3 (all *P* < 0.05). The results are shown in Table 3. For illustrative purposes, Fig. 2 shows an example of patient PET images acquired using the 6 reconstruction protocols.

**Table 3:** Subjective PET image quality scores using different reconstruction parameters (n = 63)

Parameters	OSEM3	OSEM2	HYPER0.3	HYPER0.4	HYPER0.5	HYPER0.6
*Overall image quality*						
Underweight	5.0 ± 0	5.0 ± 0	4.8 ± 0.2	5.0 ± 0	5.0 ± 0	5.0 ± 0
Normal	3.8 ± 0.6	4.7 ± 0.4	3.9 ± 0.6	4.5 ± 0.5	4.5 ± 0.5	4.5 ± 0.4
Overweight	3.1 ± 0.3	4.1 ± 0.3	3.2 ± 0.4	4.0 ± 0	4.0 ± 0.1	4.1 ± 0.4
Obese	2.7 ± 0.5	3.6 ± 0.5	3.0 ± 0	3.7 ± 0.3	3.9 ± 0.2	3.8 ± 0.3
*Noise*						
Underweight	5.0 ± 0	5.0 ± 0	5.0 ± 0	5.0 ± 0	5.0 ± 0	5.0 ± 0
Normal	3.8 ± 0.4	4.7 ± 0.4	3.9 ± 0.6	4.2 ± 0.4	4.6 ± 0.5	4.8 ± 0.4
Overweight	3.2 ± 0.4	4.1 ± 0.3	3.3 ± 0.4	4.0 ± 0	4.3 ± 0.4	4.7 ± 0.4
Obese	2.7 ± 0.5	3.6 ± 0.5	3.0 ± 0.2	3.9 ± 0.2	4.0 ± 0.0	4.3 ± 0.4
*Lesion conspicuity*						
Underweight	4.2 ± 0.3	3.6 ± 0.5	5.0 ± 0	5.0 ± 0	5.0 ± 0	4.9 ± 0.3
Normal	4.2 ± 0.3	3.5 ± 0.4	5.0 ± 0	5.0 ± 0	4.9 ± 0.2	4.7 ± 0.5
Overweight	4.1 ± 0.3	3.6 ± 0.5	5.0 ± 0	5.0 ± 0	4.9 ± 0.2	4.7 ± 0.4
Obese	4.2 ± 0.3	3.6 ± 0.4	5.0 ± 0	5.0 ± 0	4.9 ± 0.2	4.5 ± 0.5

Data are means ± standard deviations

### Lesion detectability

Table 4 shows the SUV_max_, SUV_peak_ and T/N of 94 tumour lesions in OSEM3, OSEM2, HYPER0.3, HYPER0.4, HYPER0.5 and HYPER0.6. The differences in SUV_max_, SUV_mean_ and T/N among the BMI groups were not statistically significant. The SUV_max_, SUV_peak_ and T/N of the lesions were significantly higher in HYPER0.3, HYPER0.4, and HYPER0.5 and lower in OSEM2 than in OSEM3 (all *P* < 0.001). However, there was no significant difference between OSEM3 and HYPER0.6 (all *P* > 0.05). A total of 8/94 lesions (8.5%) showed lower SUV_max_, SUV_peak_ and T/N in HYPER reconstructions than in OSEM3. The average lesion size was 1.83 cm (range, 0.8–5 cm), and the average SUV_max_, SUV_peak_ and T/N of these lesions in OSEM3 were 4.96 ± 1.07, 4.05 ± 0.89, and 2.64 ± 0.69, respectively. These lesions are located in the liver (5), colon (1), prostate (1), and celiac lymph node (1).

**Table 4:** Quantitative measurements results of lesions derived from different reconstruction parameters (n = 94)

Parameters	OSEM3	OSEM2	HYPER0.3	HYPER0.4	HYPER0.5	HYPER0.6
*SUVmax*						
Underweight	10.27 (2.86–34.89)	9.59 (2.81–34.28)	13.05 (3.05–39.91)	13.04 (3.00–39.77)	13.02 (2.95–39.65)	13.00 (2.89–39.54)
Normal	13.41 (3.22–47.01)	13.18 (2.92–46.86)	17.19 (3.26–55.01)	17.13 (3.17–55.99)	17.08 (3.12–54.99)	17.04 (3.04–54.94)
Overweight	11.35 (3.13–43.89)	10.71 (2.84–41.53)	13.87 (3.71–51.75)	13.81 (3.66–51.44)	13.76 (3.62–51.33)	13.75 (3.57–51.19)
Obese	11.64 (4.25–36.56)	10.79 (3.56–37.18)	13.67 (4.23–37.85)	13.54 (4.10–37.84)	13.42 (3.95–37.83)	13.30 (3.77–37.80)
*SUVpeak*						
Underweight	8.01 (2.57–28.92)	7.53 (2.52–28.82)	8.64 (2.67–29.40)	8.63 (2.64–29.38)	8.62 (2.61–29.36)	8.62 (2.58–29.33)
Normal	9.49 (2.81–41.58)	9.29 (2.63–41.05)	10.24 (2.85–41.13)	10.26 (2.81–41.15)	10.27 (2.78–41.15)	10.28 (2.68–41.16)
Overweight	9.18 (2.51–33.76)	8.90 (2.30–31.11)	9.58 (2.85–34.64)	9.57 (2.84–34.63)	9.56 (2.83–34.62)	9.55 (2.81–34.59)
Obese	9.34 (3.27–28.24)	8.93 (2.92–28.33)	9.89 (3.41–29.18)	9.83 (3.27–29.18)	9.79 (3.27–29.18)	9.73 (3.17–29.17)
*T/N*						
Underweight	7.91 (1.87–24.23)	7.11 (1.83–23.81)	9.50 (2.01–27.91)	9.48 (1.97–27.81)	9.46 (1.94–27.53)	9.44 (1.90–27.46)
Normal	6.81 (1.81–26.56)	6.15 (1.64–26.18)	8.21 (1.85–29.01)	8.51 (1.80–28.98)	8.49 (1.77–28.80)	8.48 (1.73–28.78)
Overweight	6.94 (1.92–19.33)	6.60 (1.73–18.21)	7.90 (2.30–22.61)	7.87 (2.26–22.56)	7.85 (2.22–22.41)	7.85 (2.19–22.45)
Obese	5.53 (1.58–16.77)	5.15 (1.40–16.82)	6.22 (1.64–17.52)	6.07 (1.62–17.44)	5.98 (1.60–17.35)	5.88 (1.57–17.26)

Data are medians (range)

**Fig. 3:**
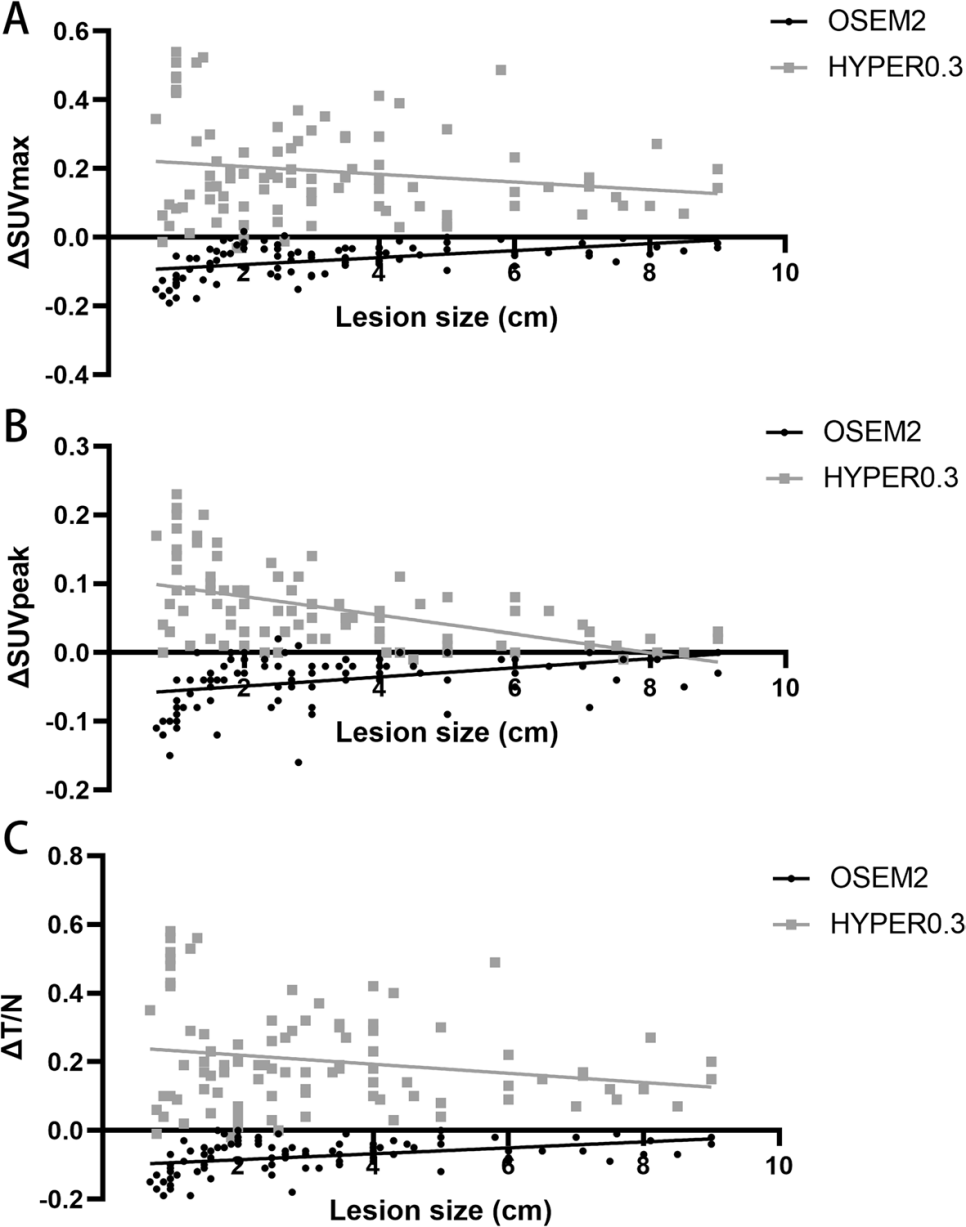
The plots showing correlation between lesion size and the variation rate of SUV_max_ (**A**), SUV_peak_ (**B**) and T/N (**C**) compared to OSEM3 in two representative groups (OSEM2 and HYPER0.3). Lines indicate the linear regression of the respective cohort. The regression trend of other groups was consistent with HYPER0.3

**Fig. 4:**
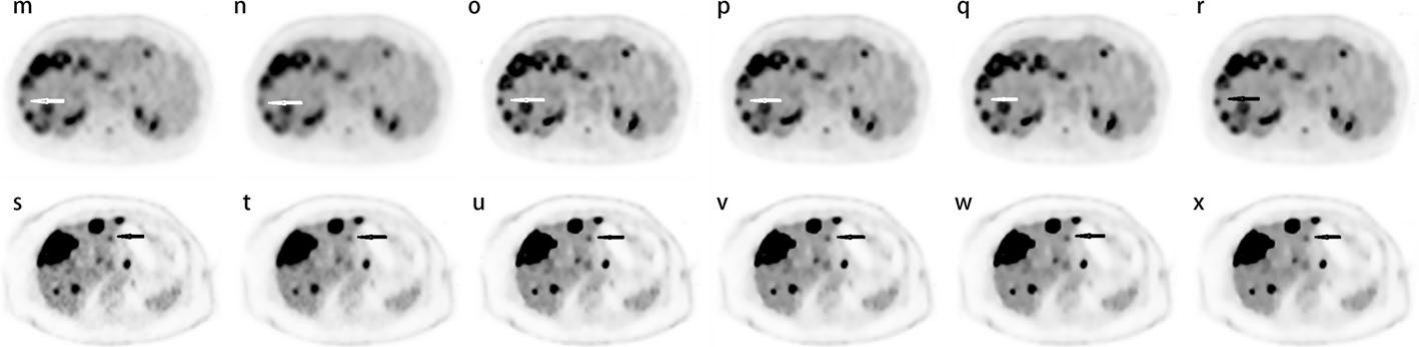
(up) Transaxial view images of a 49-year-old woman (BMI = 17.2) with metastatic colon adenocarcinoma confirmed by aspiration biopsy (m–r) for OSEM3 (**m**), OSEM2 (**n**), HYPER0.3 (**o**), HYPER0.4 (**p**), HYPER0.5 (**q**), and HYPER0.6 (**r**). The white arrows show avid FDG of the right hepatic lobe more remarkable in the HYPER reconstructions compared with OSEM reconstructions. (down) A 67-year-old woman (BMI = 30.7) with Hepatic metastasis of breast carcinoma. The FDG avid lesions were shown on the liver parenchyma in the transaxial view for the group OSEM3 (**s**), OSEM2 (**t**), HYPER0.3 (**u**), HYPER0.4 (**v**), HYPER0.5 (**w**) and HYPER0.6 (**x**). There was equal or decreased diagnostic confidence of lesion detectability (indicated by black arrows) in HYPER reconstructions compared with OSEM3(s)

The differences in SUV_max_, SUV_mean_, and T/N between OSEM2 and OSEM3, HYPER0.3 and OSEM3, HYPER0.4 and OSEM3, HYPER0.5 and OSEM3 and HYPER0.6 and OSEM3 (noted as ΔSUV_max_, ΔSUV_peak_ and ΔT/N, respectively) are listed in Table 5. The ΔSUV_max_ and ΔT/N decreased with increasing *β*-values. The median lesion size of 94 tumour lesions was 3.25 cm (range, 0.7–11.0 cm). There was a negative correlation between lesion size and ΔSUV_peak_ (*r* = 0.388, − 0.515, − 0.495, − 0.464, and − 0.423, respectively, all *P* < 0.001) (Fig. 3B). However, the correlation between lesion size and ΔSUV_max_ and lesion size and ΔT/N was not significant in the HYPER Iterative reconstructions (Fig. 3A, C). Figure 4 shows lesion visualization examples of patient PET images acquired using 6 reconstruction parameters.

**Table 5:** Correlation between lesion size and variation rate of SUV_max_, SUV_peak_ and T/N compared to OSEM3 in each group

Groups	ΔSUVmax			ΔSUVpeak			ΔT/N		
	Value	r	*P*	Value	r	*P*	Value	r	*P*
OSEM2	− 0.07 ± 0.05	0.464	< 0.001	− 0.04 ± 0.04	0.388	< 0.001	− 0.07 ± 0.05	0.407	< 0.001
HYPER0.3	0.19 ± 0.14	− 0.174	0.093	0.06 ± 0.06	− 0.515	< 0.001	0.20 ± 0.15	− 0.197	0.057
HYPER0.4	0.19 ± 0.14	− 0.158	0.128	0.06 ± 0.06	− 0.495	< 0.001	0.19 ± 0.15	− 0.182	0.080
HYPER0.5	0.18 ± 0.14	− 0.146	0.162	0.06 ± 0.06	− 0.464	< 0.001	0.18 ± 0.15	− 0.175	0.092
HYPER0.6	0.17 ± 0.15	− 0.134	0.197	0.05 ± 0.06	− 0.423	< 0.001	0.17 ± 0.15	− 0.173	0.095

## Discussion

The increasing use of ^18^F-FDG PET in oncological patients leads to increased radiation exposure of patients and medical personnel during follow-up examinations. Pursuing as-low-as-reasonably achievable (ALARA) doses has long been the work of medical imaging researchers. The straightforward advantage of the total-body PET scanner is that it reduces patient and operator exposures. Moreover, low-activity imaging benefits the development of new candidate tracers that are commonly costly. Third, increasing patient throughput with a certain amount of tracer also increases cost-effectiveness. However, imaging evaluation of the total-body with ultra-low activity injection is not yet sufficient. Patients with a wide variety of BMIs in clinical tumour FDG-PET studies may impact imaging statistics. Images of obese patients have poor quality due to photon attenuation and high scatter fractions. Our study preliminarily explored the application of OSEM reconstruction and HYPER Iterative reconstruction tools in the administered activity reduction of total-body ^18^F-FDG PET/CT over a wide range of patient BMIs. HYPER Iterative reconstructions demonstrated better lesion conspicuity and noise reduction than OSEM reconstructions especially in obese patients.

For the underweight group, OSEM and HYPER Iterative reconstructions performed excellent image quality in general. With increasing BMI, the SNR decreased significantly in OSEM reconstructions, but was relatively stable in HYPER Iterative reconstructions. This confirms the finding that BPL provides consistent liver SNR across BMI values, whereas OSEM showed decreasing SNR with increasing BMI [24]. Thus, the HYPER Iterative has a modest effect on image quality in underweight patients, and the benefit is greater for heavier individuals. Since lesion conspicuity is higher in HYPER Iterative reconstructions, the addition of HYPER Iterative reconstructions to improve lesion visibility may be considered for those with higher BMI.

Previous studies show that patients with a larger BMI consistently generate poorer image quality when using OSEM reconstruction [17, 24]. Our previous study also showed that the acceptability of the SNR_L_ should be more than 14.0 to meet the needs of image quality [25]. Consistent with the previous study, the SNR of the obese group could also could meet the need for image quality in the OSEM2 and HYPER Iterative groups with a 15-min duration, whereas OSEM3 showed image with sub-optimal noise. The image quality of OSEM2 is relatively good but with relatively poor lesion conspicuousness. The EANM procedure guidelines recommended increasing the emission acquisition time in patients weighing more than 75 kg (especially > 90 kg) to improve image quality [26]. Therefore, experienced technicians need to modify the acquisition scheme for specific situations in clinical scenarios, which is more challenging to achieve. Moreover, the extended acquisition time is intolerable for many subjects with malignancies, which may result in motion artifact. It may be appropriate to perform multiple PET reconstructions with different reconstruction settings, to maximize lesion detectability or to meet local preferences for visual interpretation of the FDG PET/CT study [26]. The HYPER Iterative algorithm adds the noise control process to each iteration, which compensates for low counts or poor-quality data. The SNR was higher and the noise was lower in HYPER Iterative reconstructions than in OSEM3. Scores of the HYPER Iterative reconstructions were also higher than that of OSEM3. However, the selection of reconstruction parameters needs to be balanced between image noise and lesion conspicuity because lesion conspicuity scores decreased with increasing *β*-values but image noise scores increased.

In our study, the SUV of lesions in HYPER Iterative reconstructions was higher than that in OSEM reconstruction. The results were consistent with the findings of a previous study using ^68^Ga-PSMA PET in which a significant increase in lesion SUV_max_ was reconstructed with HYPER Iterative [18]. ΔSUV_peak_ was correlated with lesion size which suggests that small lesions have larger SUV_peak_ elevations than large lesions. These results supported that Bayesian penalized likelihood reconstruction could improve lesion contrast, especially in small lesions [27]. To a certain extent, HYPER Iterative reconstructions improve the T/N of lesions by reducing background noise and increasing lesion SUV_max_. However, we also noticed that HYPER Iterative yields a small SUV reduction in lesions with low tracer uptake compared with OSEM3. As the *β* values increase, the image becomes smoother. The detection ability of low-FDG-uptake lesions decreased (especially in the liver). These lesions in HYPER0.6 have the propensity for missed diagnosis of lesions according to visual analysis and the minimal level of tumour uptake proposed in PERCIST 1.0 [28]. Even though the image noise of HYPER0.6 was relatively low, the tendency of missed diagnoses was unacceptable, which is not recommended for clinical practice.

There were some limitations in this study. First, it was a single-centre study, and limited lesions could not be classified and analysed in detail. Systematic analysis of more lesions is better to demonstrate the efficiency of the reconstruction algorithms. Second, this study only explored different reconstruction protocols of BMI groups. The combination of individualized acquisition time and reconstruction protocols will be further explored in future studies. Third, this study only focuses on nonclinical outcome assessments (visual assessment of image quality and SNR) in different reconstructions. Future work could investigate whether improvements in image quality and lesion conspicuity are helpful in clinical utility.

## Conclusion

Considering the image quality and lesion analysis in ^18^F-FDG total-body PET/CT with ultra-low activity injection, OSEM reconstructions with 3 iterations meet the clinical requirements in patients with BMI < 29.9. In patients with BMI ≥ 30, it is recommended that the HYPER Iterative algorithm (*β*-value of 0.3–0.5) be used to ensure consistent visual image quality and quantitative assessment.

## Data Availability

The data that support the findings of this study are available from the corresponding author upon reasonable request.

## Abbreviations

BMI: Body mass index
PET/CT: Positron emission tomography/computed tomography
OSEM: Ordered subsets expectation maximization
TOF: Time of flight
PSF: Point spread function
SD: Standard deviation
SNR: Signal to noise ratio
SUV_max_: Maximum standard uptake value
SUV_peak_: Peak standard uptake value
SUV_maean_: Mean standard uptake value
T/N: Tumour background ratio
ROI: Region of interest
NEC: Noise equivalent counts
